# Huang-Pu-Tong-Qiao Formula Ameliorates the Hippocampus Apoptosis in Diabetic Cognitive Dysfunction Mice by Activating CREB/BDNF/TrkB Signaling Pathway

**DOI:** 10.1155/2021/5514175

**Published:** 2021-06-09

**Authors:** Shu Ye, Dao-Jun Xie, Peng Zhou, Hua-Wu Gao, Meng-Ting Zhang, Da-Bao Chen, Yun-Peng Qin, Xin Lei, Xin-Quan Li, Juan Liu, Ya-Xun Cheng, Yong-Chuan Yao, Biao Cai, Guo-Ming Shen

**Affiliations:** ^1^School of Integrated Chinese and Western Medicine, Anhui University of Chinese Medicine, Hefei 230012, Anhui Province, China; ^2^Institute of Integrated Chinese and Western Medicine, Anhui Academy of Chinese Medicine, Hefei 230012, Anhui Province, China; ^3^Graduate School of Anhui, Anhui University of Chinese Medicine, Hefei 230012, Anhui, China; ^4^The First Affiliated Hospital of Anhui University of Traditional Chinese Medicine, Hefei 230031, Anhui Province, China; ^5^Anhui Province Key Laboratory of Chinese Medicinal Formula, Hefei 230012, Anhui Province, China

## Abstract

**Background:**

Huang-Pu-Tong-Qiao formula (HPTQ), a traditional Chinese medicine (TCM) formula used to improve cognitive impairment. However, the underlying neuroprotective mechanism of HPTQ treated for diabetic cognitive dysfunction (DCD) remains unclear. The purpose of this study was to investigate the neuroprotective mechanism of HPTQ in DCD mice based on molecular docking.

**Methods:**

To investigate the neuroprotective effect of HPTQ in DCD, the Morris water maze (MWM), novel object recognition (NOR) test was used to detect the learning and memory changes of mice; hematoxylin-eosin (HE) staining was used to investigate the damage of hippocampal neurons; the western blot (WB) was used to examine the level of brain-derived neurotrophic factor (BDNF) of hippocampus. To investigate the neuroprotective mechanism of HPTQ in DCD, molecular docking was used to predict the possible target proteins of different active components in HPTQ and then the WB was used to verify the expression of key target proteins in the hippocampus of mice.

**Results:**

HPTQ improved the learning and memory ability, hippocampal neuron damage, and the level of BDNF in the hippocampus of the DCD model treated with HFD/STZ for 12 weeks. Besides, the results of molecular docking showed that the main chemical components of HPTQ could be well combined with the targets of Bcl-2-associated X (Bax) and B-cell lymphoma2 (Bcl-2) and caspase-3. The levels of Bax/Bcl-2 protein ratio and caspase-3 increased in the DCD model while the HPTQ inhibited it. In addition, HPTQ restored DCD-induced decline of p-CREB, BDNF, TrkB, and p-Akt in the hippocampus.

**Conclusions:**

These data indicated that HPTQ ameliorates the hippocampus apoptosis in diabetic cognitive dysfunction mice by activating CREB/BDNF/TrkB signaling pathway.

## 1. Introduction

Diabetic cognitive dysfunction (DCD), a diabetic central nervous system (CNS) complication, is characterized by cognitive deficits, neurochemical electrophysiological neurostructural abnormalities [1–3]. Without control of glycemic imbalance in CNS, it is considered to be the initiating factor in the development of it [4]. The hippocampus is an important part of the CNS, participates in the regulation of various nervous systems, and plays a fundamental role in memory and learning [5]. It is sensitive to fluctuations in glucose levels, some studies showed that glucose administration could improve hippocampal function and cognitive ability [6], but chronic hyperglycemia might increase the impact of dementia [7]. In addition, the hippocampus involved in the pathophysiology of streptozocin (STZ) induced cognitive dysfunction [8, 9]. The loss of glucose levels in the hippocampus may lead to mitochondrial dysfunction, initiate apoptosis, and eventually lead to hippocampal neuron damage. The pathogenesis of DCD is complex, including insulin signaling disorders, autonomic dysfunction, and neuroinflammatory pathway abnormalities, advanced glycation end products deposition, mitochondrial metabolism disorders, etc. [10]. Currently, the underlying neuroprotective mechanism of this disorder has not been elucidated. The prolonged occurrence of DCD may have a negative impact on the patient's social life.

Huang-Pu-Tong-Qiao (HPTQ) is a hospital formula from the *First Affiliated Hospital of Anhui University of Traditional Chinese Medicine*, which has been used in the clinical treatment of dementia for many years. It is composed of six herbal medicines, including seven chemical compositions, mainly Stilbene glycoside, ferulic acid, ginsenoside Rg1, aloe-emodin, *β*-asarone, emodin, and chrysophanol [11]. Pharmacological studies have shown that the main extract components of HPTQ, such as ginsenoside Rg1 and Tetrahydroxystilbene glucoside, might improve cognitive impairment of Alzheimer's disease (AD) animal models in multiple pathways [12, 13]. Our previous studies have demonstrated that HPTQ could improve cognitive impairments of AD rats by inhibiting multiple pathways, including EGFR-PLC*γ*, CaM-CaMKIV, oxidative stress, and mitochondrial apoptotic signaling pathways [11, 14]. The HPTQ could also improve the apoptosis of primary hippocampal neurons induced by A*β*_25-35_ [15]. However, due to the complex composition of HPTQ, the potential efficacy and mechanism for treating DCD have not been elucidated.

In this study, we first established the DCD mice model and investigated the neuroprotective effects and potential mechanism of HPTQ based on molecular docking.

## 2. Materials and Methods

### 2.1. Animals

Male C57BL/6 mice (10 weeks old, weighing 20–22 g) were purchased from Qinglongshan Animal Breeding Farm. (Nanjing, China) Certificate no. SCXK (Su) 2017–0001. All experiments animals were housed at room temperature (25 ± 3°C) and humidity (55–70%) under a light/dark cycle of 12 h/12 h, with free access to water and standard diets. The study protocol was approved by the Animal Management Center of the Anhui University of Chinese Medicine, China.

### 2.2. Experimental Induction of DCD Model and Treatments Schedule

After excluding animals with outlying results by the Morris water maze (MWM) test, the mice were randomly divided into two groups: the control group (CTRL, *n* = 20) and the diabetes mellitus group (DM, *n* = 40). DM (or DCD) group was induced by a mix of high-fat diet (HFD) and low dose streptozotocin (STZ, St. Louis, MO, USA). DM (or DCD) group were fed with HFD (formula: 15% lard, 10% sucrose, 3% cholesterol, 3% sodium chloride, 69% standard rat feed) for 4 weeks and were administrated with streptozotocin (STZ) by intraperitoneal (i. p.) at 55 mg/kg (dissolved in 100 mM sodium citrate buffer pH = 4.5) for 5 consecutive days [16]. The CTRL group was administrated with an equivalent volume of citrate buffer. Seven days after the STZ was administrated, the fasting blood glucose (FBG) levels more than 16.7 mmol/L were considered as diabetic mice and the blood was collected from the tail vein. Body weight was measured weekly and FBG every two weeks. The MWM test was used to detect the changes of learning and memory ability at the 0, 4th, 8th, 12th, and 16th week and the brain-derived neurotrophic factor (BDNF) was detected at the same time.

**Figure 1:**
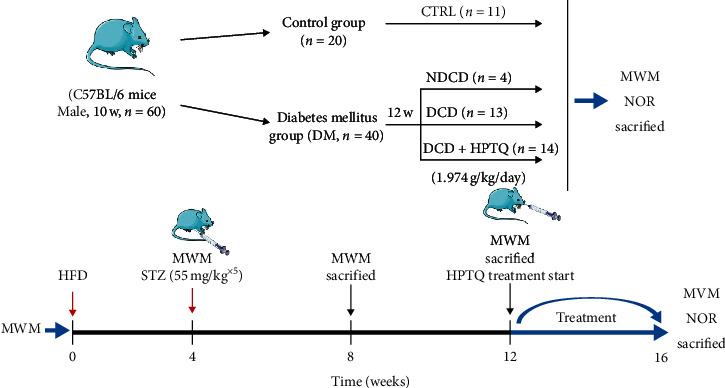
Scheme of experimental designs. HFD: high-fat diet; MWM: morris water maze; NOR: novel object recognition.

When the MWM test screened diabetic mice for cognitive impairment, the mice were randomly divided into three groups **(**Four mice did not make the DCD model, NDCD): Control group (CTRL group, *n* = 11), diabetic cognitive dysfunction (DCD group *n* = 13), and Huang-Pu Tong-Qiao formula intervention group (HPTQ group *n* = 14). The HPTQ group mice were treated with HPTQ (1.974 g/kg) for 4 weeks. After 4 weeks of administration by gavage, MWM and novel object recognition test were performed to assess the cognitive functions, and then the next experiment was carried out. The schedule for this test was shown in Figure 1.

### 2.3. Behavioral Experiments

#### 2.3.1. Morris Water Maze Test

The spatial learning and memory ability of mice was detected with MWM. The MWM was a circle pool (diameter: 120 cm; height: 55 cm), filled with a depth of 30 cm water mixed with milk powder. The pool was divided into four equal spaced quadrants and then a hidden platform (diameter: 8 cm) was placed 1 cm below the water in one of the quadrants. Spatial training was conducted for 5 consecutive days. The mice were put into the water from each quadrant in turn and the time is taken to find the platform within 90 s was recorded. If the mice did not find the platform within 90 s, they were placed on the platform and left there for 15 s. On the fifth day, without guidance, the duration for finding the platform was recorded as escape latency. On the sixth day, the platform was removed, allowing each mouse to swim freely for 90 seconds inside the pool. The number of times that the mice crossed the area (where the platform was removed) was recorded by a video camera.

#### 2.3.2. Novel Object Recognition Test

The novel object recognition (NOR) test was performed for assessing cognition, based on the principle that animals have the instinct to explore new objects [17]. The test consists of three phases: habituation phase, training phase, and test phase. The test apparatus was composed of a rectangular white box (25 × 25 × 40 cm) and three objects named A, B, and C, of which A was the same as B, while the object of C was completely different in shape and colour from the A and B. The test is based on previous research with some modifications [18]. The first day was the habituation phase, and mice were acclimated to the box for 5 min in the absence of objects, then returned to the mouse cage. The next day was the training phase; mice were exposed to A and B (placed in opposite corners of box 3 cm away from the walls) and each mouse was allowed to explore for 5 min. 24 hours later, the B was replaced with C, and mice were also placed back in the box to explore freely for 5 minutes. Exploration was defined as the nose to the object at a distance within 2 cm or touching it with the nose. The box and objects were cleaned with 70% ethanol between trials to avoid residual odors. The relative exploration time of the novel object (TN) and the familiar object (TF) was recorded. The preference index = (TN − TF)/(TN + TF).

### 2.4. Preparation of Tissue Samples

After the behavioral test, the mice were euthanized with 1% sodium pentobarbital (40 mg/kg). The hippocampus tissues were quickly separated on ice and stored at −80°C for western blot experiments.

### 2.5. Determination of Insulin Level in Hippocampus

The frozen hippocampus tissue samples were cleaned by precooling phosphate buffer saline (PBS) and then put into a glass homogenizer to add the corresponding volume of PBS for full grinding. After centrifugation for 10 minutes at 3000 r/min, the supernatant was detected by an insulin ELISA kit provided by Elabscience Biotechnology Co., Ltd (Elabscience, Wuhan, China).

### 2.6. Hematoxylin-Eosin Staining (HE)

The mice were sacrificed by anesthesia with an intraperitoneal injection of 1% sodium pentobarbital (40 mg/kg). The brain samples were fixed overnight with 4% formaldehyde and then embedded in paraffin. The 5 mm thick coronal sections of brain samples were dewaxed and dyed with HE. The hippocampal neurons in the CA1 region were observed with an Olympus X51 microscope.

### 2.7. Molecular Docking

CB-Dock predicts cavities of the protein and calculates the centers and sizes of the top N (*n* = 5 by default) cavities. PDB formats of caspase-3 (PDB Code: 1GFW) [19], Bax (PDB Code: 4S0O) [20], Bcl-2 (PDB Code: 2W3L) [21], and ligand files of in SDF formats of the main chemicals in HPTQ were input to CB-Dock to elevate the binding activities [22, 23].

### 2.8. Western Blot

The total protein was extracted from hippocampus tissue using a radio-immunoprecipitation assay (RIPA) buffer containing protease inhibitors (Abmole biotechnology, Shanghai, China). The 20 *μ*g extracted proteins were separated by 10% SDS-PAGE (Bio-Rad) and transferred onto polyvinylidene fluoride (PDVF) membrane (Millipore, Billerica, MA, USA). The membrane was blocked with 5% nonfat dry milk for 2 h and incubated overnight at 4°C with the relevant primary antibodies. The primary antibodies were anti-BDNF(1 : 1000, novus biologicals, Beijing, China), anti-caspase-3 (1 : 1000, Affinity, Liyang, China), anti-Bax (1 : 1000, Abcam, Cambridge, UK), anti-Bcl-2 (1 : 1000, Abcam, Cambridge, UK), anti-p-CREB (1 : 1000, Cell Signaling Technology, MA, USA), anti-TrkB(1 : 1000, Cell Signaling Technology, MA, USA), and anti-p-Akt (1 : 2000, Cell Signaling Technology, MA, USA). The membrane was incubated with the horseradish peroxidase (HRP)-conjugated goat anti-rabbit immunoglobulin G (IgG) (1 : 20,000, Abbkine, Wuhan, China). The membrane was visualized by the proteins by ECL reagent and analyzed by ImageJ software. Rabbit anti-rat *β*-actin (1 : 2000, Abbkine, Wuhan, China) was used as an internal control.

### 2.9. Statistical Analysis

The results were expressed as means ± standard deviation (SD). Data were analyzed by a one-way ANOVA test and followed by Tukey's multiple comparison test for comparison of two groups. *P* < 0.05 was considered statistically significant.

## 3. Results

### 3.1. The Establishment of DCD Animal Model

To establish the mice model of DCD, we measured the body weight, the fasting blood glucose, the learning and memory, and the level of BDNF at different stages.

**Figure 2:**
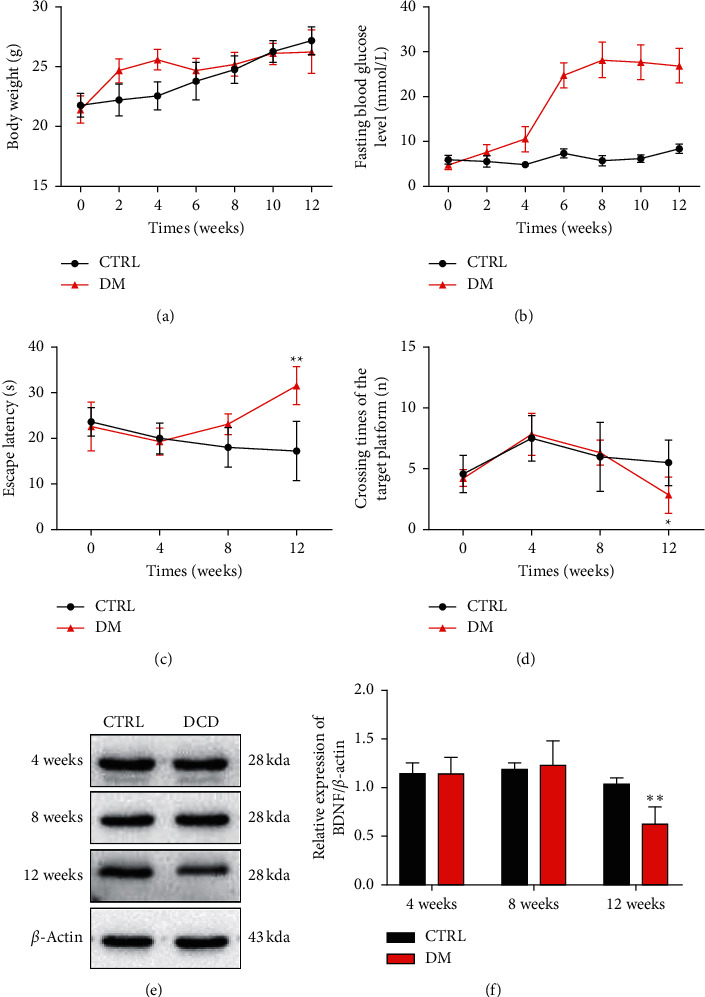
The establishment of DCD animal model. Body weights in different periods (a), fasting plasma glucose levels in different periods (b). Escape latency for escaping to a platform in the 5-day consecutive training trials (c). The crossing times of target platform (d). Expression of BDNF in the hippocampus of mice in different periods (e, f). Values are in mean ± SD. ^*∗*^*P* < 0.05, ^*∗∗*^*P* < 0.01, vs. the CTRL group.

The body weight of mice with HFD was significantly increased, but the level of blood glucose changed not obviously; two weeks after the STZ administrated, the mice exhibited severe hyperglycemia in the DM group, blood glucose assessment identified a total of 40 mice developed DM, the body weight of in different groups mice increased continuously, but the difference was not statistically significant (Figures 2(a) and 2(b)).

The MWM test was used to investigate the learning and memory of mice in different time periods. From the first week to the eighth week, there were no significant changes in escape latency and times of crossing platform tests in different groups. In the 12th week, the escape latency of the DM group was signally prolonged and the number of times the DM group crossed the platform was signally decreased compared with CTRL (Figures 2(c)–2(d)). The level of BDNF in the hippocampus was determined with western blot. Compared with the CTRL group, the level of BDNF was significantly lower in 12 weeks (Figures 2(e)–2(f)).

The MWM and the level of BDNF assessment identified a total 27 out of the 31 HFD/STZ mice developed DCD. The results have shown that 12 weeks of HFD/STZ intervention, the DCD model was successfully established in C57BL/6 mice. The remaining mice were then excluded from the further study, which was divided into two groups: diabetic cognitive dysfunction (DCD group) and Huang-Pu-Tong-Qiao formula intervention group (HPTQ).

### 3.2. Effect of HPTQ on Weight, Blood Glucose, and Insulin Levels of DCD Model

**Figure 3:**
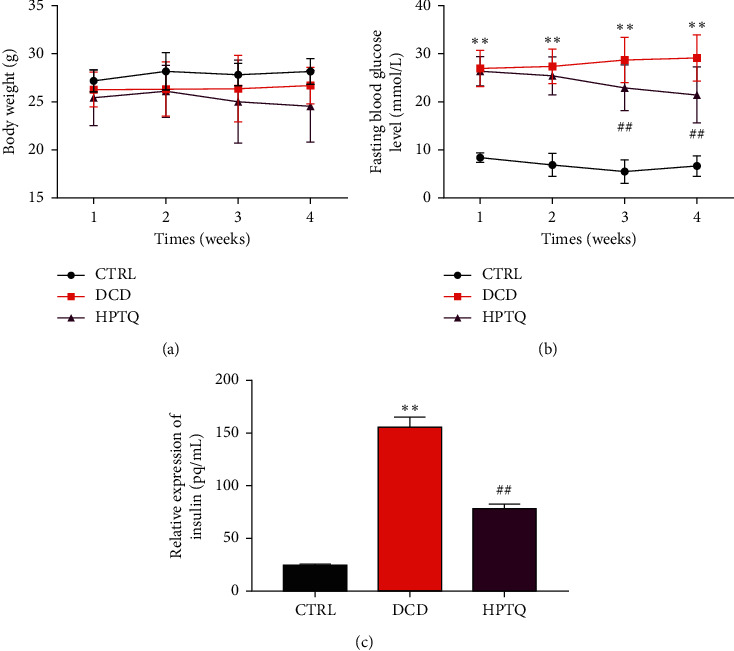
Effect of HPTQ on weight, blood glucose, and insulin levels of DCD model. Body weights (a), fasting plasma glucose levels (b), insulin levels (c), and values are mean ± SD. ^*∗*^*P* < 0.05, ^*∗∗*^*P* < 0.01, vs. the CTRL group. ^#^*P* < 0.05, ^##^*P* < 0.01, vs. the DCD group.

To investigate the metabolic changes of HPTQ in DCD, the weight, blood glucose, and insulin levels in the hippocampus were detected. The body weight of mice in the CTRL and DCD group has been a tendency toward rising, but there is no statistical significance; after HPTQ intervention, the weight loss trend of mice in the HPTQ group was slower than that in the DCD group, but there was no significant difference between two groups (Figure 3(a)). The fasting blood glucose and insulin levels of DCD group mice were elevated continuously, compared with the CTRL group; after intragastric administration of HPTQ for 28 days, the fasting blood glucose and insulin levels of HPTQ group mice decreased significantly, compared with the DCD group (Figures 3(b)–3(c)).

### 3.3. HPTQ Improved Learning and Memory in DCD Mice

**Figure 4:**
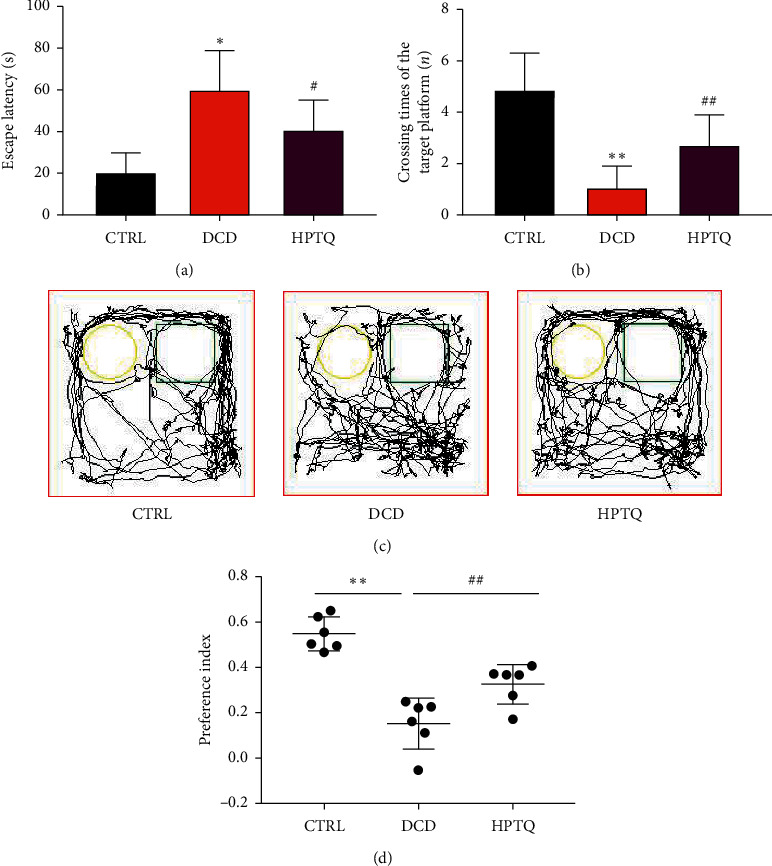
HPTQ improved learning and memory in DCD mice. The escape in the hidden platform (a), the crossing times of target platform (b), the trajectory of mice in NOR (c), and the preference index in DCD mice (d), the preference index (time spent at the novel object-time spent at the familiar object)/(time spent at the novel object + time spent at the familiar object)]. Values are means ± SD. ^*∗*^*P* < 0.05, ^*∗∗*^*P* < 0.01 vs. CTRL; ^#^*P* < 0.05, ^##^*P* < 0.01.

To investigate whether HPTQ could relieve memory and learning impairments, we performed the tests of Morris water maze and novel object recognition. In the MWM test, the time for DCD group mice to find target platform was signally increased and the number of times crossed the platform was signally decreased compared with the CTRL group (Figure 4(a)); after being treated with HPTQ, the time of mice to find target platform was signally decreased and the number of times it crossed the platform was signally increased compared with the DCD group (Figure 4(b)). As an alternative method to confirm whether HPTQ ameliorates diabetes-induced memory dysfunction, we used the NOR test, which is based on the principle that animals have the instinct to explore new objects. In the NOR test, the preference index of the DCD group mice was significantly lower than that of the CTRL group. However, the preference index in HPTQ group mice improved compared to the DCD group (Figures 4(c)–4(d)).

### 3.4. Effects of HPTQ on Hippocampal Neurons in CA1 Region of DCD Mice

**Figure 5:**
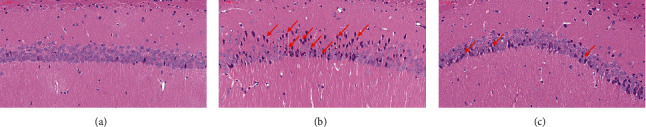
Effects of HPTQ on hippocampal neurons in the CA1 zone of DCD mice. CTRL group (a), DCD group (b), and HPTQ group treated with dose of HPTQ (1.974 g/kg) (c).

HE staining was used to observe the damage of hippocampal neurons in the CA1 region. As shown in Figure 5, CTRL group neuron's structure was round closely arranged and pale-stained nuclei (Figure 5(a)); DCD group neuron's structure was disordered notably shrunken, atrophied, and irregularly arranged, indicating that injury induced significantly neurons deteriorated and damaged compared with CTRL group in hippocampal CA1 area (Figure 5(b)). However, the neuronal damage was significantly improved after treatment with HPTQ in DCD, indicating that HPTQ might have a neuroprotective effect (Figure 5(c)).

### 3.5. Molecular Docking

**Figure 6:**
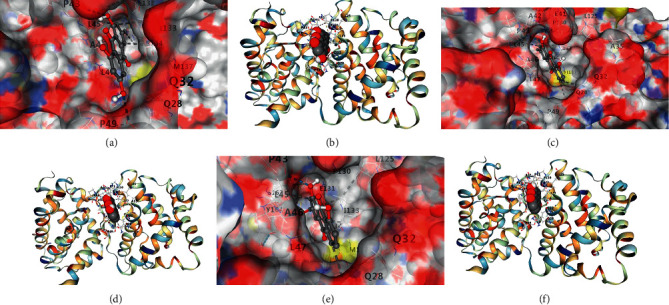
Docking of main components of HPTQ with Bax. Emodin docked with Bax (a, b), chrysophanol docked with Bax (c, d), and aloe-emodin docked with Bax (e, f).

**Figure 7:**
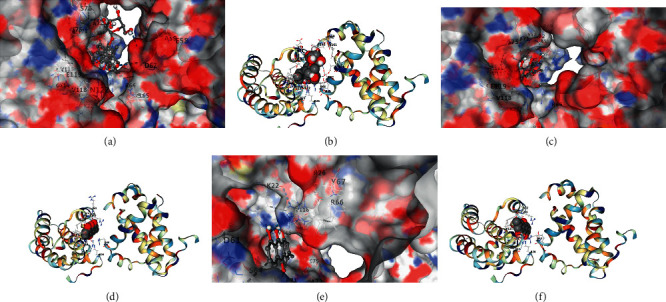
Docking of main components of HPTQ with Bcl-2. Ginsenoside Rg1 docked with Bcl-2 (a, b), aloe-emodin docked with Bcl-2 (c, d), and chrysophanol docked with Bcl-2 (e, f).

**Figure 8:**
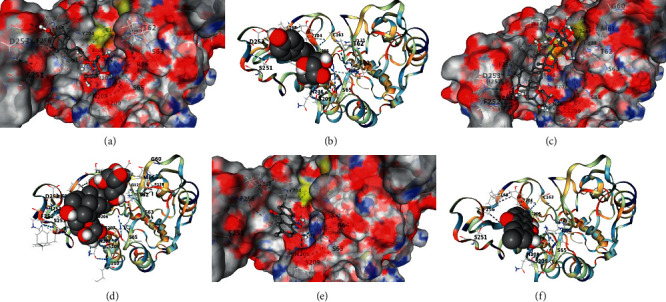
Docking of main components of HPTQ with caspase-3. Stilbene glycoside docked with Caspase-3 (a, b), finsenoside Rg1 docked with Caspase-3 (c, d), and chrysophanol docked with caspase-3 (e, f).

Molecular docking results showed that the main components of HPTQ have a good binding ability with Bax, Bcl-2, and caspase-3. In our docking results, emodin has the strongest binding ability with Bax, followed by chrysophanol and aloe-emodin (Table 1, Figures 6(a) and 6(f)); ginsenoside Rg1 has the strongest binding ability to Bcl-2, followed by aloe-emodin and chrysophanol (Table 2, Figures 7(a)–7(f)); Stilbene glucoside has the strongest binding ability to caspase-3, followed by ginsenoside Rg1 and chrysophanol (Table 3, Figures 8(a)–8(f)).

### 3.6. Effect of HPTQ on Apoptosis-Related Proteins in DCD Model

**Figure 9:**
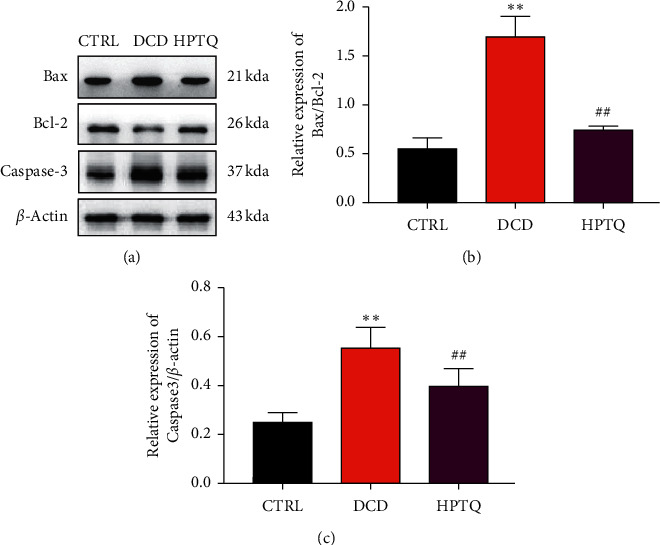
The protein levels of Bax, Bcl-2, and caspase-3 in hippocampal neurons. Bax, Bcl-2, and caspase-3 protein levels in hippocampus neurons were measured by WB (a). The protein loading intensity was calculated with *β*-actin as internal (b, c). Values are means ± SD. ^*∗*^*P* < 0.05, ^*∗∗*^*P* < 0.01 vs CTRL group; ^#^*P* < 0.05, ^##^*P* < 0.01 vs DCD group.

Based on previous research study and molecular docking results, the expression of Bax, Bcl-2, and caspase-3 was determined by western blot to further validate underlying the protective effect of HPTQ in the DCD model. Compared with the CTRL group, the protein expression of Bax/Bcl-2 and caspase-3 in the DCD group was higher significantly. When treated with HPTQ, the Bax/Bcl-2 and caspase-3 level in the HPTQ group were lower significantly than those in the DCD group (Figures 9(a) and 9(b)).

### 3.7. Effect of HPTQ on Antiapoptosis Related Proteins in DCD Model

**Figure 10:**
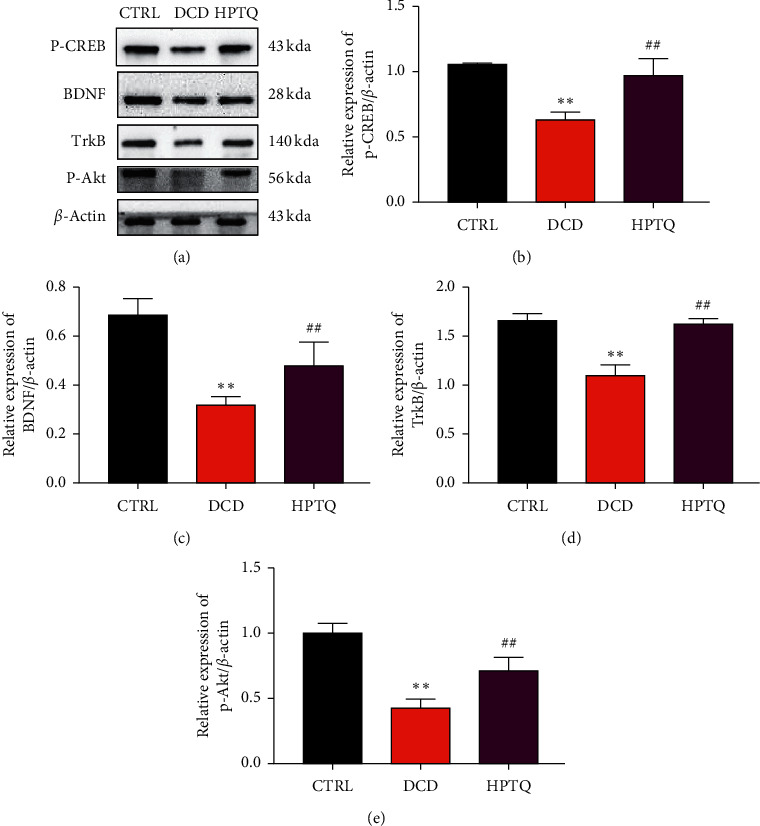
The protein levels of p-CREB, BDNF, TrkB, and p-Akt in hippocampal neurons. p-CREB, BDNF, TrkB, and p-Akt protein levels in hippocampus neurons were measured by WB (a). The protein loading intensity was calculated with *β*-actin as an internal control (b–e). Values are means ± SD. ^*∗*^*P* < 0.05, ^*∗∗*^*P* < 0.01 vs CTRL group; ^#^*P* < 0.05, ^##^*P* < 0.01 vs DCD group.

In order to further validate the mechanism of HPTQ in DCD. The protein levels of p-CREB, BDNF, TrkB, and p-Akt in hippocampal neurons were detected by WB. The result showed that compared with the CTRL group, the level of p-CREB, BDNF, TrkB, and p-Akt were significantly decreased in the DCD group. However, compared with the DCD group, the protein expression of p-CREB, BDNF, TrkB, and p-Akt were significantly increased (Figure 10) in the HPTQ group.

## 4. Discussion

A large number of epidemiological studies showed that diabetes mellitus is prone to cognitive decline and dementia [24, 25], whether it is type 1 diabetes or type 2 diabetes. In recent years, there are more and more researches on DCD, but the exact mechanism is not clear.

Previous studies have shown that a high-fat diet combined with streptozotocin (HFD/STZ) could successfully establish a DCD animal model, but the dosage and use time of STZ are different [16, 18]. Based on this, a high-fat diet combined with low dose STZ was used to replicate the natural history and metabolic characteristics of human type 2 diabetes mellitus. In order to establish a stable DCD model, the time points of cognition impairment after HFD/STZ modeling were screened out. We used the MWM to detect the cognitive changes once every 4 weeks and the expression of BDNF in the hippocampus at the same time point. The MWM results showed that the escape latency increased, and the times of crossing the platform decreased significantly in the 12^th^ week. The WB results showed that the level of BDNF decreased significantly in the 12^th^ week. However, we did not find any significant difference of morphological in the hippocampal region between the different groups in the first 12 weeks. It confirmed that C57BL/6 mice suffered cognitive impairment in 12 weeks from behavioral and molecular biology. Hippocampus is vulnerable to hyperglycemic, prolonged high glucose induced neuronal damage [26]. The BDNF level was measured as a brain-derived neurotrophic function marker in DCD mice hippocampus. It is an essential modulator in CNS, supporting neuronal survival and promoting the growth and differentiation of new neurons [27].

Currently, the treatments for diabetes have failed to prevent the decline in cognitive function, so alternative treatment strategies are urgently needed. Traditional Chinese medicine (TCM) compound has the advantages of multicomponent and multitarget, so it has gradually become a new research hotspot. HPTQ is mainly composed of six natural herbal medicines, including a variety of chemical components, showing multicomponent and multitarget. Our previous study documented the positive effects of HPTQ in cognitive impairment [11]. 28 days after HPTQ administration, it abolished the enhanced insulin level in the hippocampus of mice with DCD, indicating that HPTQ could facilitate the efficiency of hippocampal insulin in DCD mice. Notably, HPTQ decreased the escape latency considerably, increased the times of crossing platform, and improved the preference index of DCD mice, implying that HPTQ could improve hippocampal-mediated learning and memory abilities of DCD mice. In addition, it significantly improved the damage of hippocampal neurons in DCD mice and upregulated the protein expression of BDNF, which may be an important reason for HPTQ to enhance memory.

To further investigate the preventive effect of HPTQ on the progression of DCD, molecular docking was performed. The results of molecular docking showed that the main chemical components of HPTQ could be well combined with the targets of Bax, Bcl-2, and caspase-3, indicating that HPTQ may act on the targets of Bax, Bcl-2, and caspase-3. Bax, Bcl-2, and caspase-3 are all mitochondria-related apoptosis proteins. As reported, mitochondria are central regulators of neurons, which are damaged as key factors underlying cognitive defects in neurodegenerative diseases [28]. Bax is a proapoptotic factor and Bcl-2 is an antiapoptotic factor, which localizes on the mitochondrial surface, play an important role in the process of apoptosis [29, 30]. Following overexpression of Bax, it moves from the cytosol to the outer mitochondrial membrane and forms homo-oligomeric complexes with Bax, resulting in the changes in the permeability of the mitochondrial outer membrane. Disorders of mitochondrial metabolism can result in the release of a variety of proapoptotic factors, activate caspase 3 or caspase 7, and initiate apoptosis [31, 32]. The hippocampus plays an important role in the learning and memory process. Therefore, targeting hippocampal neurotrophy may be a potential strategy to reduce the risk of DCD and improve DCD outcomes. To further investigate the preventive effect of HPTQ on DCD, with the support of molecular docking technology, the level of mitochondria-related apoptosis proteins Bax, Bcl-2, and caspase-3 in hippocampal tissue were performed. Our results have revealed that the levels of caspase-3 and the ratio of Bax to Bcl-2 were significantly increased in the DCD mice model, confirming that severe apoptosis occurred via the mitochondria-related Bax/Bcl-2 and caspase-3 pathway. However, after treatment with HPTQ, it downregulated levels of caspase-3 and the Bax/Bcl-2.

**Figure 11:**
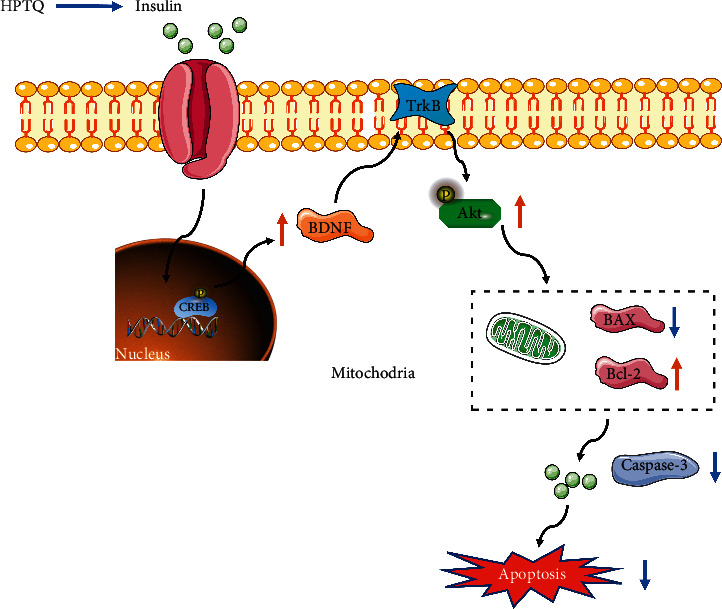
Presentation of mechanism for the neuroprotective role of HPTQ in DCD mice model. HPTQ could reduce the release of insulin, activate CREB/BDNF/TrkB signaling pathway, and inhibit mitochondria-related apoptosis signals Bax/Bcl-2 and caspase-3 pathway in the brain hippocampus.

BDNF plays an important role in the pathophysiology of mitochondrial dysfunction [33]. Similar to BDNF, transmembrane protein receptor tropomyosin receptor kinase B (TrkB) is widely expressed in CNS. BDNF elicits regulatory roles in multiple neuroprotective effects by binding with TrkB that further activates its downstream signaling pathways including/protein kinase B(PI3K/Akt) [34–36]. PI3K/Akt pathway is particularly important in widely mediating survival signals [37]. Phosphorylation of Akt promotes cell survival and differentiation and reduces neuronal apoptosis [38]. Activation of TrkB by BDNF induces the phosphorylation of Akt [39]. cAMP response element-binding protein (CREB), a transcription factor located in the nucleus, is involved in glucose homeostasis [40]. The levels of CREB and the active form of p-CREB were decreased in the rodent models of insulin resistance and insulin deficiency diabetes [41]. Furthermore, a previous study revealed that CREB phosphorylation could upregulate the expression of BDNF [42, 43]. The combination of BDNF and TrkB activates related intracellular signaling pathways, thereby increasing hippocampal neuron synaptic activity, promoting neurogenesis, reducing apoptosis, and improving spatial learning and memory [42, 43]. It has been demonstrated that activation of BDNF/TrkB could inhibit Bax/Bcl-2 ratio and further inhibit caspase-3 [44]. Our study elucidated that the levels of p-CREB, BDNF, TrkB, and p-Akt were decreased in DCD mice and upregulated after HPTQ intervention. Furthermore, our findings elucidated that HPTQ markedly suppressed the enhancement of Bax/Bcl-2 ratio and activation of caspase-3, suggesting that HPTQ could attenuate the injury of a hippocampal neuron, improve the ability of learning and memory in the DCD mice model through activating CREB/BDNF/TrkB signaling pathway and inhibiting mitochondrial apoptosis pathway (Figure 11).

## 5. Conclusions

In conclusion, our data demonstrated that after 12 weeks of HFD/STZ intervention, the DCD model was successfully established in C57BL6 mice. Simultaneously, chronic HPTQ treatment debilitated diabetes-induced cognitive deficits and regulated hippocampal insulin metabolism activated CREB/BDNF/TrkB signaling pathway and suppressed the mitochondria-related Bax/Bcl-2 and caspase-3 apoptosis pathway. The antiapoptotic effect of HPTQ may be through control the level of insulin and activate CREB/BDNF/TrkB signaling pathway in the DCD mice brain. These findings indicated that HPTQ might be considered as a potential therapeutic agent for diabetic cognitive dysfunction. However, further evidence is still needed to confirm this phenomenon.

## Figures and Tables

**Table 1:** Docking of main components of HPTQ with Bax.

Chemicals	Vina score	Cavity score	Center (*x*, *y*, *z*)	Size (*x*, *y*, *z*)
*Emodin*	−8.5	316	17, −9, 14	19, 19, 19
*Chrysophanol*	−8.4	316	17, −9, 14	19, 19, 19
*Aloe-emodin*	−8.2	316	17, −9, 14	20, 20, 20
Ginsenoside Rg_1_	−7.7	316	17, −9, 14	27, 27, 27
Stilbene glycoside	−6.9	316	17, −9, 14	20, 20, 20
Ferulic acid	−6.3	316	17, −9, 14	19, 19, 19
*β*-asarone	−5.7	316	17, −9, 14	18, 18, 18

**Table 2:** Docking of main components of HPTQ with Bcl-2.

Chemicals	Vina score	Cavity score	Center (*x*, *y*, *z*)	Size (*x*, *y*, *z*)
*Ginsenoside Rg*_*1*_	−9.6	673	38, 35, 2	27, 27, 27
*Aloe-emodin*	−7.9	673	38, 35, 2	20, 20, 20
*Chrysophanol*	−7.9	673	38, 35, 2	19, 19, 19
Emodin	−7.8	673	38, 35, 2	19, 19, 19
Stilbene glycoside	−7.2	673	38, 35, 2	21, 21, 21
Ferulic acid	−5.7	673	38, 35, 2	20, 20, 20
*β*-asarone	−5.2	673	38, 35, 2	18, 18, 18

**Table 3:** Docking of main components of HPTQ with caspase-3.

Chemicals	Vina score	Cavity score	Center (*x*, *y*, *z*)	Size (*x*, *y*, *z*)
*Stilbene glycoside*	−7.7	271	37, 35, 32	21, 21, 21
*Ginsenoside Rg*_*1*_	−7.2	271	37, 35, 32	27, 27, 27
*Chrysophanol*	−7.1	271	37, 35, 32	19, 19, 19
Emodin	−6.9	271	37, 35, 32	19, 19, 19
*β*-asarone	−6.7	271	37, 35, 32	18, 18, 18
Aloe-emodin	−6.6	271	37, 35, 32	20, 20, 20
Ferulic acid	−5.8	271	37, 35, 32	20, 20, 20

## Data Availability

The data will be made available upon reasonable request.

## Supplementary Materials

Supplementary Materials Tables S1–S6: the raw data about the establishment of the DCD model and therapeutic effect of HPTQ on DCD mice.
