# Supplementary material for: Huang-Pu-Tong-Qiao Formula Ameliorates the Hippocampus Apoptosis in Diabetic Cognitive Dysfunction Mice by Activating CREB/BDNF/TrkB Signaling Pathway
Source: Evid Based Complement Alternat Med. 2021 Jun 9;2021:5514175. doi: 10.1155/2021/5514175 (PMC8211510; doi:10.1155/2021/5514175)
Supplement: Supplementary Materials — Tables S1–S6: the raw data about the establishment of the DCD model and therapeutic effect of HPTQ on DCD mice. [file 5514175.f1.pdf]

Table S1 The levels of body weight and fasting plasma glucose at different time in CTRL group and DM group mice before DCD model

Groups	CTRL	weight	FBG	DM	weight	FBG
0weeks	1	21.5	4.3	1	21	4.6
	2	21.6	3.9	2	23.4	4.5
	3	23.5	5.3	3	20	6.7
	4	21.4	6	4	21.7	4.5
	5	20.5	4.6	5	20.9	4.2
	6	22.2	6.1	6	21.5	4.5
2weeks	1	20.5	5.3	1	24.9	8.3
	2	24.1	5.2	2	24.5	5.3
	3	21.2	6.1	3	25.3	5.2
	4	21.7	4.8	4	24.1	6.7
	5	22.8	6.1	5	26	6.8
	6	23.1	6.3	6	23.1	6.3
4weeks	1	20.4	4.3	1	25.3	9.4
	2	23	5.3	2	25.3	12.8
	3	22.2	5.1	3	26.2	14.3
	4	23	6.9	4	25.6	9.4
	5	23.8	4.9	5	26.8	6.4
	6	23	5.6	6	24.3	10.8
6weeks	1	21	7	1	24.8	20
	2	24.1	8.7	2	24	26.4
	3	24.6	8.3	3	25.2	24.6
	4	25.5	7	4	24	25.1
	5	23.1	7.3	5	26.4	28.3
	6	24.5	5.9	6	23.6	24.2
8weeks	1	22.6	6.4	1	25.4	23.1
	2	25.8	4.8	2	24.4	29.8
	3	25.49	6.8	3	26.1	33
	4	24.7	6.2	4	24.7	29.5
	5	24.7	3.8	5	26.6	30
	6	25.3	6.3	6	24.1	23.6
10weeks	1	24.5	6.4	1	26.7	23.1
	2	27.1	5.8	2	25.7	26.8
	3	26.6	6.8	3	26.9	33
	4	26.5	7.2	4	25.9	29.5
	5	26.3	4.8	5	26.7	30
	6	26.6	6.3	6	24.5	23.6
12weeks	1	25.4	7.7	1	26.3	26.1
	2	28.9	9.1	2	24.6	29
	3	27.7	8.3	3	25.5	30
	4	27.4	9	4	28.9	20.2
	5	26.7	6.8	5	24.4	25.8
	6	27	9.5	6	27.9	30.3

Table S2 The results of water maze test at different time in CTRL group and DM group mice before DCD model establishment

Groups	CTRL	Escape latency (s)	Crossing times of the target platform (n)	DM	Escape latency (s)	Crossing times of the target platform (n)
0weeks	1	22.86	3.5	1	24.42	3.5
	2	21.72	4	2	26.52	4.5
	3	26.1	7.5	3	30.26	5
	4	28.12	4	4	16.26	4
	5	23.5	3.5	5	19.26	3.5
	6	19.3	5	6	18.82	5
4weeks	1	22.78	5	1	15.02	7
	2	25.18	6	2	21.42	6
	3	16.38	8	3	22.46	9
	4	18.06	9	4	16.38	6
	5	20.22	10	5	20.38	9
	6	17.34	7	6	20.14	10
8weeks	1	24.06	11	1	21.86	6
	2	14.46	4	2	21.42	7
	3	14.94	5	3	23.58	6
	4	16.62	7	4	22.86	8
	5	14.94	3	5	21.5	6
	6	22.86	6	6	27.42	5
12weeks	1	21.16	3	1	28.8	5
	2	15.44	7	2	28.36	4
	3	6.2	4	3	37.56	3
	4	16.28	5	4	27.32	1
	5	18.92	6	5	35.56	2
	6	25.48	8	6	31.8	2

Table S3 Effect of HPTQ on the level of weight and blood glucose in DCD mice

Groups	CTRL	weight	FBG	DM	weight	FBG	HPTQ	weight	FBG
1week	1	25.4	7.7	1	26.3	26.1	1	29.5	29.2
	2	28.9	9.1	2	24.6	29	2	27.6	30.1
	3	27.7	8.3	3	25.5	30	3	24.1	23.4
	4	27.4	9	4	28.9	20.2	4	22.6	25.1
	5	26.7	6.8	5	24.4	25.8	5	22.2	27.7
	6	27	9.5	6	27.9	30.3	6	26.7	22.8
2weeks	1	25.4	7.7	1	29.9	22.1	1	25.3	30
	2	28.9	7.1	2	26.4	29	2	30.3	29.5
	3	29.7	8.3	3	25.8	32	3	26.5	25.3
	4	27.4	3	4	22.5	30	4	25.3	25.8
	5	30.7	9.8	5	29.2	25.8	5	22.1	22.2
	6	27	5.5	6	24.2	25.3	6	27.1	19.9
3weeks	1	27.4	5.1	1	30.8	28.4	1	29	29.2
	2	28.9	2.1	2	29.2	20.5	2	30.6	28.7
	3	29.7	5.5	3	22.4	30	3	25.3	21.9
	4	27.4	6.2	4	22.8	33.3	4	20.2	19.4
	5	26.7	4.7	5	27.9	27.1	5	20.2	18.7
	6	27	9.6	6	25.2	33	6	24.9	19.8
4weeks	1	28	4.9	1	29	30	1	22.7	19.4
	2	29.6	7.1	2	25.4	33	2	30.7	29.4
	3	29.9	9.4	3	25.1	20.5	3	20.2	25
	4	27.6	3.6	4	28	33.3	4	22	24.4
	5	26.5	6.7	5	24.5	27.2	5	25.9	16.1
	6	27.3	8.1	6	28.1	30.9	6	25.8	14.3

Table S4 Effect of HPTQ on the learning and memory in DCD mice model tested by morris water maze

CTRL	Escape latency (s)	Crossing times of the target platform (n)	DCD	Escape latency (s)	Crossing times of the target platform (n)	HPTQ	Escape latency (s)	Crossing times of the target platform (n)
1	5.44	3	1	85.24	1	1	49.24	4
2	34.84	7	2	69.24	0	2	35.64	1
3	18.44	4	3	51.64	0	3	32.04	2
4	24.44	5	4	29.64	1	4	65.24	3
5	15.64	6	5	68.84	2	5	23.32	4
6	21.24	4	6	53.24	2	6	36.44	2

Table S5 Effect of HPTQ on the learning and memory in DCD mice model tested by novel object recognition

CTRL	Preference Index	DCD	Preference Index	HPTQ	Preference Index
1	0.504	1	0.2273	1	0.1738
2	0.468	2	0.1629	2	0.3674
3	0.556	3	0.1127	3	0.3714
4	0.494	4	0.222	4	0.3681
5	0.652	5	0.25	5	0.2771
6	0.625	6	-0.051	6	0.4063

Table S6 Effect of HPTQ on the level of insulin in DCD mice

CTRL	insulin	DCD	insulin	HPTQ	insulin
1	26.1565	1	151.3739	1	77.89565
2	24.5043	2	154.3304	2	78.50435
3	22.2435	3	147.4609	3	83.89565
4	22.7652	4	169.7217	4	73.89565
